# Ethics-sensitivity of the Ghana national integrated strategic response plan for pandemic influenza

**DOI:** 10.1186/s12910-015-0025-9

**Published:** 2015-05-07

**Authors:** Amos Laar, Debra DeBruin

**Affiliations:** Department of Population, Family and Reproductive Health, School of Public Health, College of Health Sciences, University of Ghana, Box LG 13, Legon, Accra, Ghana; Center for Bioethics, University of Minnesota, 410 Church Street S.E MN, 55455-0346 Minneapolis, USA

**Keywords:** Pandemic preparedness plan, Ethics, Ethics sensitivity, Developing country, Ghana

## Abstract

**Background:**

Many commentators call for a more ethical approach to planning for influenza pandemics. In the developed world, some pandemic preparedness plans have already been examined from an ethical viewpoint. This paper assesses the attention given to ethics issues by the Ghana National Integrated Strategic Plan for Pandemic Influenza (NISPPI).

**Methods:**

We critically analyzed the Ghana NISPPI’s sensitivity to ethics issues to determine how well it reflects ethical commitments and principles identified in our review of global pandemic preparedness literature, existing pandemic plans, and relevant ethics frameworks.

**Results:**

This paper reveals that important ethical issues have not been addressed in the Ghana NISPPI. Several important ethical issues are unanticipated, unacknowledged, and unplanned for. These include guidelines on allocation of scarce resources, the duties of healthcare workers, ethics-sensitive operational guidelines/protocols, and compensation programs. The NISPPI also pays scant attention to use of vaccines and antivirals, border issues and cooperation with neighboring countries, justification for delineated actions, and outbreak simulations. Feedback and communication plans are nebulous, while leadership, coordination, and budgeting are quite detailed. With respect to presentation, the NISPPI’s text is organized around five thematic areas. While each area implicates ethical issues, NISPPI treatment of these areas consistently fails to address them.

**Conclusions:**

Our analysis reveals a lack of consideration of ethics by the NISPPI. We contend that, while the plan’s content and fundamental assumptions provide support for implementation of the delineated public health actions, its consideration of ethical issues is poor. Deficiencies include a failure to incorporate guidelines that ensure fair distribution of scarce resources and a lack of justification for delineated procedures. Until these deficiencies are recognized and addressed, Ghana runs the risk of rolling out unjust and ethically indefensible actions with real negative effects in the event of a pandemic. Soliciting inputs from the public and consultation with ethicists during the next revision of the NISPPI will be useful in addressing these issues.

## Background

During the last century, the world’s population experienced one devastating influenza pandemic and three less severe global outbreaks. First was the Spanish influenza in 1918 (A/H1N1); the Asian influenza occurred in 1957 (H2N2); and the Hong Kong influenza in 1968 (H3N2) [1,2]. The Swine flu pandemic occurred in 2009 (HIN1/09) [3]. Even though no public health expert can predict with certainty the timing and severity of the next outbreak, there is general agreement that future pandemics are perhaps inevitable [4].

With this understanding, the World Health Organization (WHO) periodically publishes recommendations for countries to use in their own preparations. Previous ones include “the role of WHO and recommendations for national measures before and during pandemics” [5], and a checklist for influenza preparedness, both released in 2005 [6]. In 2006, the WHO released a protocol for rapid response and containment [7], and a draft protocol for rapid response and containment of pandemic influenza in 2007 [8]. Thenceforth, earnest preparations for an influenza pandemic have been proceeding worldwide. The level of preparedness, however, varies from place to place.

For example, in November 2005 the US laid out a broad national strategy for pandemic response [9]. This was followed by a more detailed pandemic influenza plan in 2006 [10]. In the state of Minnesota for example, the legislature authorized $4.085 million in 2007 to improve the state’s preparedness for pandemic influenza. The Minnesota Department of Health was advised to spend $3.97 million of this funding to purchase antiviral medication and to stockpile medical supplies [11].

Preparations in resource-constrained settings are usually thin, focusing on public health actions and goals. Little if any attention is given to ethics considerations. We use the Republic of Ghana’s pandemic preparedness plan as an illustrative example. The Ghana National Integrated Strategic Plan for Pandemic Influenza for 2009 – 2013 (NISPPI) is the government’s paper outlining its preparedness strategies [12]. The NISPPI addresses five main themes - planning and coordination; surveillance and situation monitoring; prevention, containment and management; communication; and social mitigation. In the recent past, the plan has been critiqued for deficiencies in epidemiological content and other concerns [13,14]. Sambala criticizes the vaccine/antiviral strategies of Ghana, Malawi, and Tanzania pandemic plans as unclear and inadequate – lacking an epidemiological explanation as to why certain groups are at high risk. Norman et al. do not consider the level of risk used in estimating direct health threat in the event of an outbreak acceptable risk. They do not offer what level of risk is acceptable. Both the 2006 influenza preparedness and response plan [15] and the current NISPPI [12] contain assumptions about the direct health threat in the event of an outbreak. Both project the number of clinical cases and deaths in Ghana in the event of a Phase 5 pandemic^a^ using gross attack rates of between 15% and 40%, and case fatality rates (CFR) between 0.6% and 1.5%. Based on Ghana’s present population of approximately 25 million, even these conservative assumptions show a real potential for significant mortality (22,500 deaths) in an influenza pandemic. Clinical cases will far exceed what the country’s health infrastructure and sophistication can handle.

Furthermore, the overall cost of actions deemed necessary to prepare the health care delivery system to respond to human avian influenza was estimated to be about $11,000,000 [12]. The actual amount that was released to relevant ministries, departments and agencies (MDAs) is unknown but is guessed to be an insignificant fraction.

Aside from these general challenges, we hypothesized that the NISPPI would be deficient in ethics content. Hyder et al. [16] have noted that, while ethics is gradually being integrated into public health policy in the developed world, ethical considerations are often undervalued or even ignored by the public health policies of developing countries.

Arguments for the inclusion of ethics in pandemic planning have been made in the past, albeit primarily in relation to plans from the developed world. Jaro Kotalik offers an ethical analysis of the pandemic plans of Canada, the United Kingdom and the United States [17]. Thompson et al. [18] proposed an ethical framework for pandemic influenza planning, but their framework does not reflect developing world realities. Thomas et al. analyzed the US federal and state plans for evidence of ethical guidance [19]. Patel et al. proposed a framework, which they used to appraise pandemic plans from Australia, England, USA, New Zealand and Canada [20]. DeBruin et al., drawing on their experiences from the public engagement process of Minnesota Pandemic Ethics Project, share strategies on how to achieve social justice goals in pandemic response [21].

Papers that have considered preparedness plans from resource poor settings [13,14,22,23] have not adequately addressed ethics issues. In the developing world, ethical issues seem to be ignored in public health programming and even in scholastic discourse. Lisa Eckenwiler argues that, in the absence of explicit ethical analysis, preparedness policies are unlikely to deal adequately with ethical issues – underscoring the importance of ethical review in every pandemic preparedness planning process [24].

Taking a cue from the warning of Thomas et al. that “history will judge our generation’s response to the next pandemic in large part by our ability to act ethically” [19], our paper aims to first assess how sensitive the Ghana NISPPI is to ethics matters. Second, by highlighting the negative implications of such insensitivity on real people in the event of a pandemic, the paper initiates a debate on the utility of including ethics in pandemic preparedness.

## Methods

### Assessment of ethics sensitivity of the NISPPI

We assessed how sensitive the Ghana NISPPI is to ethics issues by critically analyzing it to determine how well it reflects ethical commitments and principles identified in our review of the global pandemic preparedness literature, existing pandemic plans, as well as relevant ethics frameworks. We also drew on lessons from SARS, and the 2009-influenza pandemic. See Table 1 for a summary of our review of the literature, highlighting ethical issues addressed therein.

**Table 1:** **Ethics sensitivity parameters**

**#**	**Ethics sensitivity parameter**	**Definition**	**Source**
	*Decision making process:*	Preparedness decision-making is deliberative and all-inclusive, with public consultations. Decisions are publicly defensible, which means that decisions are open to scrutiny and the basis upon which decisions are made are publicly accessible to affected stakeholders.	[18,32]
	*Justification for delineated actions:*	All planned actions/activities are adequately explained and justified	[17,23]
	*Composition of national pandemic planning committee:*	Committee formation process is deliberative and composition is all-inclusive with members from national, regional, and district levels; membership include technical and lay person	[23,32]
	*Communication to at risk population/Information symmetry:*	Efforts are outlined in the plan to keep the public continuously informed on all aspects of the planned interventions. Differential messages for various audiences are developed and are culturally and scientifically appropriate, pandemic phase alerts are incorporated into the communication machinery.	[17,23,43]
	*Prioritization, and allocation of scarce resources:*	Triage systems for priority setting in providing critical care, or for allocation of scarce resources (vaccines, intensive care units, hospital beds, human resource allocation, and staffing) during a pandemic are clearly outlined. Plan establishes priority groups nuanced by local contexts	[17,32,43-45]
	*Healthcare worker duty to care adequately explained:*	Plan addresses healthcare worker duty to provide care in pandemic circumstances. That is the issue of special obligations of health care professionals during an outbreak. Are the rights and responsibilities of health care professionals especially in the context of pandemic flu preparedness clarified?	[17,32,43,46]
	*Limitations of proposed interventions:*	Plan frankly acknowledges limitations of various proposed actions or interventions (both pharmaceutical and non-pharmaceutical)	[42]
	*Leadership and coordination*	Plan indicates which agencies will lead various components/actions of the plan	[23,32]
	*Facilities designation*	Facilities where patients would be treated are clearly identifies or designated	[23]
	*Stockpiling of antiviral, vaccines and personal protective equipment:*	Provisions are made for national stockpiling of antivirals, vaccines, and personnel protective equipment; and such provisions are sensitive to locale-specific competing demands	[17,23,43]
	*Ethics training for healthcare workers*	Plan adequately outlines pandemic-specific ethics training for various categories of people working in healthcare setting	[23]
	*Timelines for planned activities:*	Timelines are explicitly defined for all activities outlined in the plan	[23]
	*Border issues,* travel advisories, and trade policies	Plan explicitly and adequately addresses cooperation with neighboring countries.	[23,32,43]
	*Co-operation with the WHO other development partners, and sister countries*	Plan acknowledges the essence of cooperating with these institutions	[23,32]
	*Ethics consideration in clinical protocols:*	Plan-specific operational guidelines/protocols are developed and are ethics-sensitive	[23]
	*Use of vaccines and antivirals:*	Guidelines are issued on how antiviral, vaccines, personal protective equipment should be used during an outbreak (different from allocations). Logistical infrastructure for rapid distribution of stockpiled antivirals, vaccines, and personal protective equipment are in place.	[23,32,43]
	*Plan review mechanism:*	Plan explicitly states a mechanism for continuous review and updates	[23]
	*Continuity of essential services:*	There is evidence of planning to ensure continuity of essential services including non-health sector essential services such as ensuring business continuity, capacity for corpse disposal etc.	[43]
	*Considerations of equity, social justice, vulnerable groups:*	Plan addresses special needs of vulnerable and disadvantaged groups	[21]
	*Home care management of infected patients:*	Plan recommends home care management of infected patients, and provides guidelines	[23]
	*Social solidarity considerations:*	Plans on how to manage complex social spaces are described, importance of social “solidarity” during a pandemic is acknowledged and communicated	[18,43]
		Solidarity requires: good, open and honest communication, open collaboration, in a spirit of common purpose, within and between health care institutions, sharing public health information, coordinating health care delivery, transfer of patients, and deployment of human and material resources	
	*Plan adaptability/flexibility:*	Outlined actions are flexible and encourage evidence-informed modification as required. That is opportunities to revisit and revise decisions as new information emerges throughout the crisis as well as mechanisms to address disputes and complaints are there.	[18,43]
	*Preventive ethics consideration*	Preparedness actions ably balances emergency ethics and preventive ethics	[46]
	*Budgeting considerations:*	Plan has a budget and sources of funding are indicated	[23]
	*Outbreak simulations:*	Outbreak simulations are incorporated into preparedness activities	[23]
	*Back up essential personnel*	The need for identification and recruitment of additional essential personnel during pandemic outbreak is acknowledged and planned for	[23]
	*Ethical framework for the plan:*	There is a specific ethical framework developed to guide the plan’s implementation	[18,19,47]
	*Privacy considerations*	Plans acknowledges individual rights to privacy, and provides justifications for privacy rights bridging	[23,32]
	*Proportionality of response:*	Guidance exists in the plan to ensure responses to threat are proportional and measured. That is restrictions to individual liberty are carefully thought through and measures taken to protect the public from harm do not exceed what is necessary to address the actual level of risk to, or critical need of, the community.	[18,43]
	Compensation programs	There are mechanisms in place to ensure that ethical decision-making is sustained throughout the crisis, and measures to cater for the social and economic cost of poultry destruction, market closure, e.t.c. are outlined.	[18,22,48]

## Results and discussion

### The framework for preparedness and response to pandemic influenza in Ghana

Developed using a framework recommended by the WHO [25], and thus organized into five thematic areas, Ghana’s NISPPI aims to improve on earlier preparedness and response structures and mechanisms. Ghana has in the past responded to threats from Severe Acute Respiratory Syndrome (SARS) and Avian Influenza by producing response guidelines, albeit basic ones [15]. The five thematic areas of the current framework are summarized and discussed.

#### Planning and coordination

The planning and coordination component of the plan aims to provide high-level political support for the activation and implementation of the national integrated strategic plan for pandemic influenza. It promises involvement and commitment of all sectors, and provision of resources for efficient operationalization of the plan. The plan is tiered at three levels: the first is the national coordinating committee, the second tier is the national technical coordinating committee and the third, regional and district committees. The Ghana National Disaster Management Organization (NADMO) leads the National Coordinating Committee (NCC). The relevant MDAs identified in the plan are Ministries of Interior, Finance, Health, Food and Agriculture, Transport and Communications, Information, Employment and Social Welfare, Local Government and Rural Development, Defense, Environment Science and Technology. The Resident Coordinator of the UN System and a conglomeration of international state and non-state actors referred to as development partners are also recognized as partners in planning and coordination.

A careful reading of the NISPPI, however, reveals that some important planning and coordination actions are either not covered at all or not adequately addressed. These relate to decision making processes, justification for delineated actions, membership of pandemic planning committees, allocation of scarce resources, timelines for planned activities, border control and co-operation with development partners, among others.

Plans elsewhere [9,10,26] and pandemic-specific ethical frameworks [19,20,27,28] encourage that preparedness decision making be deliberative and all-inclusive, with public consultations [29], and that all planned actions including social distancing and other restrictions on individual liberties be adequately explained and justified. These issues are not explicitly addressed in NISPPI. Guidance on allocating scarce resources (vaccines, intensive care units, hospital beds, human resource allocation, and staffing) or triage systems for priority setting in providing critical care during a pandemic are absent from the plan. It is generally encouraged that pandemic plans establish priority groups nuanced by local contexts [30]. The other required planning actions that are not addressed include clear indication of agencies to lead the coordination and maintenance of essential services, and designation of facilities where patients should be treated in the event of a pandemic.

Ethics training particularly for healthcare workers is another essential planning action [31]. The current plan does not outline any pandemic-specific ethics training for people working in health care facilities. The plan does not appear to address cooperation with neighboring countries. Ethics-sensitive operational guidelines/protocols are absent, and so are pandemic plan review and update mechanisms. Feedback and planned communication plans were nebulous. However, we praise the plan for including an estimated budget and for acknowledging the importance of cooperating with the WHO and other development partners.

The question of who gets to make key decisions in a national public health emergency is an important ethical consideration. In an open democratic society, the development of socially significant approaches such as pandemic preparedness plans ought to follow democratic and deliberative processes. As Jaro Kotalik argues, fairness requires that those who will be affected have a say in the decision making process [17]. Thus in planning, provisions need to be made for input from various groups such as ordinary community members, healthcare workers, bioethicists, and public policy advisors, among others [27,29]. A careful reading of the NISPPI reveals that its development did not employ deliberative procedures of community engagement. All the contributors to the plan were technical personnel from various ministries, departments and agencies. These included the Ghana Health Service, the Ministry of Health, Veterinary Services/Ministry of Food and Agriculture, Wildlife Division/Ministry of Lands, Forestry and Mines, Noguchi Memorial Institute for Medical Research of the University of Ghana, NADMO, Ministry of the Interior, WHO, FAO, United States Agency for International Development (USAID), and Quality Health Partners. This process denied the ordinary Ghanaian a voice in the development of the plan. There were no public hearings.

Pandemic preparedness plans should incorporate feedback into the development process. According to available global guidelines [32], (1) all stakeholders should be represented during pandemic preparedness meetings; (2) these meetings should occur at different levels (national, regional and local) for both health and non-health sectors; (3) overarching coordinating teams should be present at meetings; and (4) effective communication channels should be established across sectors and among stakeholder levels. Both intra- and inter-sectoral cooperation and coordination are encouraged. Incorporation of a communication plan and multiple plan revisions and adaptations improve the feedback process. Last, but most important, information about the planning process and the people/organizations involved in it should be communicted to the public. The current NISPPI lacks these provisions.

#### Surveillance and situation monitoring

The surveillance and monitoring component of the plan aims to build national, regional and district capacity for early detection of and response to pandemic influenza. Strengthening of laboratory capacity for virus characterization in addition to rapid confirmation of suspected cases of pandemic influenza are mentioned as actionable strategies. Also mentioned, without elaboration under this section, is the provision of guidance on how to develop policies for antivirals and vaccine. The plan designates the Ministry of Health’s Disease Surveillance Department as coordinator of all surveillance activities. The plan mandates the Ghana Health Service to employ its structures from community through to the national levels to investigate and respond to any suspected cases of human influenza.

This section fails to provide ethics justifications for proposed actions. Moreover, the plan is silent on cooperation with neighboring countries, even though border control is an essential surveillance action. While cooperation with the WHO and other development partners are valuable, this should not be a substitute for delineation of surveillance-specific border control guidance in the NISPPI.

#### Communication

Drafters of the NISPPI envisaged that communication initiatives will increase the general public’s awareness and understanding of pandemic influenza and preparedness plans. Communications strategies can, for example, promote behavior change to reduce the risk of transmission. Pandemic plans should ensure coordinated and consistent communications at all times between authorities in all sectors and with the public. The NISPPI establishes a communication sub-committee with the Ministry of Information as the lead agency. Senior level representatives from Ministry of Information, NADMO, the Media, National Commission on Civic Education (NCCE), Health Promotion Departments of MOH/GHS and WHO are listed as members of the yet to be established committee.

However, the NISPPI outlines no measures to keep the public informed on planned interventions and justifications for them. Pre-pandemic development of differential messages for various audiences (e.g. poultry handlers, teachers, religious authorities, villagers, healthcare providers, market women, etc.) would foster more effective communication, but the NISPPI does not address such strategies. There is also no mention of pandemic phase alerts in the communication section of the plan.

Pandemic influenza plans, by their nature, must pay considerable attention to communication needs among various levels of government and related institutions [33]. Authors of the plan also note that its success will rest on clear and consistent communication about the pandemic and its risks to the Ghanaian population. Drafters of the NISPPI feel it is reasonable to initiate communication process only when an outbreak occurs. The principles of transparency and accountability require that those who are going to be affected be informed not only during the pandemic but be engaged from the development of the preparedness plans [27,29]. Civic engagement and fair processes are requirements in every phase of pandemic preparedness planning. Lessons from existing guidelines and frameworks [19,20] show that effective communication among health care professionals and the public could be achieved by integrating communication aspects into all planning, preparedness and response activities. For instance, key institutions or spokespersons for disseminating information to the public need to be identified prior to the pandemic and they need to give clear, consistent and balanced messages. Plans for evaluating how information is received and perceived by health care providers, the public and other stakeholders need to be included in the NISPPI.

#### Prevention, containment, management and social mitigation

The objectives of the prevention, containment, and management component of the plan are stated as follows: to reduce the risk of animal to human transmissions; to reduce the risk of co-infections in humans to minimize opportunities for virus re-assortment that could generate lethal strains of influenza virus; and to stockpile antivirals, vaccines, protective equipment and other logistics for efficient deployment. The Ministry of Health and the Ghana Health Service are designated as the responsible agencies.

The goal of social mitigation is to enable functioning of key systems and services (e.g. utilities, health delivery, security) during various phases of the pandemic by encouraging business continuity planning by both public and private sector agencies. Voluntary home quarantine of members of households with confirmed or probable influenza case(s) will be promoted. The NISSPI states that antiviral medications will be offered to those quarantined for prophylactic use. The plan addresses the use of social distancing as a measure to reduce contacts between individuals in the community and workplace. Cancellation of large public gatherings and suspension of markets are options. NADMO is designated to lead the humanitarian response, and to be supported by the Ghana Red Cross Society (GRCS), ministry of food and agriculture (MOFA), the Nutrition Department of the Ghana Health Service, and the MOH. Ghana’s development partners, the UN System, private sector, and NGOs are mentioned as partners. With respect to resources needed to implement the plan, the NISPPI identifies the Government of Ghana and five development partners (USAID, FAO, WHO, UNDP, EU) as sources of resources and/or technical assistance.

The NISPPI fails to provide an ethical framework to justify and guide these interventions. Moreover, allocation of scarce resources and healthcare worker duty to care, are either not mentioned or poorly addressed in the plan.

One of the chief ethical issues raised by both the 1918 influenza pandemic and also by the SARS outbreak in 2003 was the recurring tension in public health between the rights of individual liberties versus public health promotion [34]. Questions surrounding isolation, quarantine, and application of police power are relevant in this context. Even though the NISSPI mentions that quarantine and other standstill measures will be invoked immediately when the Minister declares an outbreak, the plan does not address the ethical questions associated with such interventions. Available records from Colonial Ghana amplify the need for such ethical questions to be addressed. The records indicate that social distancing methods including segregation were attempted; not only did officials close schools and ban public meetings, they also restricted the police and clerical workers from doing their duties [35]. The towns of Larwa, Tumu, and Wa in the Upper West Region of current Ghana clearly abused patients by removing them from their homes and isolating them to inhabitable huts on the fringes of the infected villages, and the larger towns and villages were completely sectioned off [36]. Food, water, and necessary supplies could only be placed outside the camps and only the attendants could come out to collect these items for the patients inside. In the northern territories and Ashanti, the Colonial administration constructed fences around infected towns, placed markets outside their borders, and directed traffic to alternative trade routes [36]. Some commentators on the subject have argued that if individuals are required to forgo certain liberties, fairness should be a prime consideration [34]. Nowhere in the NISPPI is this issue addressed.

Second, the NISSPI’s plan to stockpile antiviral agents or vaccines is charged with ethical conundrums. Questions such as the basis for decisions to stockpile antiviral agents versus procuring medical supplies needed for immediate use are ethically relevant. Stockpiling of antiviral agents is a key part of national influenza pandemic preparedness in many industrialized countries [26]; it is also a dream for pandemic plans in resource-constrained settings. The current NISSPI has cost estimates on procuring pharmaceuticals and other supplies. This is estimated to be $709,750. It is reasonable to expect that a justification for this expenditure be provided when a significant proportion of the country’s population is in serious need for basic medical supplies such as antimalarial and antiretroviral drugs.

How are ethical issues associated with triaging and allocation of scarce resources during a pandemic dealt with in the NISSPI? Many countries, especially developing countries, will be forced to confront the next pandemic with few or no available vaccines or other resources. According to global estimates, a pandemic, if severe, could lead to too many sick people all over the world, all requiring care at the same time [4]. Should this happen in Ghana, the already inadequate human and material resources of the Ghana health services will be further and rapidly overstretched. Based on experiences elsewhere, many of the sick will recover with minimal assistance, but others will be seriously ill and require prolonged hospitalization, diagnostic facilities, multiple drugs, and aid from health personnel if they are to have a chance to survive [17]. Even though Ghana would not have enough antivirals and related resources, the NISSPI does not recognize the need to provide guidelines on how to ethically allocate very scarce resources during a pandemic.

The NISSPI provides no guidance to address the ethical issue of health care worker duty to care in pandemic circumstance. Every pandemic plan should address the question of whether practitioners are obligated to treat patients with a highly contagious disease, thus putting themselves and their families, acquaintances, or anyone else they contact at risk. For example, research publications have mentioned the high transmissibility to nurses and frontline physicians in Southeast Asia during the SARS outbreak [37,38]. Wenzel et al. report that about 50% of those who died from SARS were healthcare workers who had come in contact with infected patients in hospitals [38]. Chua et al. recorded that fear and anxiety-induced psychiatric morbidity was a major problem among healthcare workers involved in SARS treatment [39]. Drawing from these experiences, others have warned that an influenza pandemic will impose similar foreseeable risks to physicians and other healthcare workers [40]. This raises an ethical dilemma about the extent of the professional duty of caregivers during a pandemic versus the limits to health risk to themselves and to their families that healthcare workers need accept [41]. It may be argued that if provided with the appropriate equipment or supplies necessary to treat, practitioners should be obligated to treat someone with any illness. The NISPPI mentions procuring and distributing protective clothing, masks, aprons, and gloves. This is laudable; however, being prepared requires not only that health workers are aware that they have masks that they can use during a pandemic but also that they have the requisite training on how to use personal protective equipment safely and effectively. Indeed, it is required of planners to acknowledge limitations of various proposed actions or interventions (both pharmaceutical and non-pharmaceutical) [42]. The NISPPI does not address these issues.

### Recommendations

We applaud the organization of the text of the NISPPI around the WHO’s five thematic areas. However, each of these five themes -- planning and coordination; surveillance and situation monitoring; prevention, containment and management; communication; and social mitigation – are infused with ethical issues. Unfortunately, NISPPI does not attend to these issues as it plans in relation to the themes. We recommend that the Ghana Ministry of Interior, Ministry of Health and their partners incorporate ethics into their pandemic preparedness efforts. This could be done by introducing a running sixth theme on ethics. This ethics theme could be applied more systematically to the five categories - acting as a lens on the other five categories. Seeking public input and consultation with ethicists during plan development or revisions will prove useful in addressing these deficiencies.

Although all the ethics deficiencies identified are relevant, we would prioritize efforts that aim at improving the decision making process, providing guidance on healthcare worker duty to care, and nuanced stockpiling of and prioritized allocation plans for antiviral, vaccines and personal protective equipment. We especially flag planning actions that contribute to equity, social justice, and respect for vulnerable groups as important. Preparedness decision-making could be made deliberative and all-inclusive through public and stakeholder input and consultation with ethicists. Adequate public and stakeholder engagement would ensure that the stockpiling and allocation of resources are sensitive to locale-specific competing demands. The plan must address special needs of vulnerable and disadvantaged groups and institute measures to ensure that responses to threat are proportional and measured.

### Limitations of the analysis

The findings of this analysis are subject to a number of limitations, which are worth noting. First, given the volatility of the local public health, somewhat time-fixed plans may exist alongside other national or sub-national guidelines undergoing constant revisions. Such guidelines, if they do exist, did not form part of this analysis. The second limitation relates to the fact that the quality of ethical argumentation in preparedness plans and actual amount of attention ethics will receive in an influenza pandemic are distinct. Though an important indicator of her sensitivity to ethics, the quality or otherwise of emphasis to ethics in the NISPPI is one among many elements. We note therefore that our analysis of plans is an incomplete but important assessment of the ethics sensitivity of Ghana’s preparedness for influenza pandemic.

## Conclusions

Notwithstanding the many strengths of the NISPPI in terms of its public health content, guidance on how to address ethical issues during a pandemic remains nascent. Lack of guidelines to ensure fair allocation of scarce resources during a pandemic, failure to justify proposals for procuring and stockpiling pharmaceuticals and other medical supplies, and failure to justify delineated preparedness actions are deficiencies. Until the deficiencies are recognized and addressed, Ghana runs the risk of rolling out unjust and ethically indefensible actions with real negative effects on people in the event of a pandemic threat. Soliciting input from the public and consultation with ethicists during next revision of the NISPPI will be useful in addressing these issues.

## Endnote

^a^A scenario that would result from the world progressing to Phase 5 of the pandemic with the appearance of a viral strain capable of rapid and effective human-to-human transmission. In this scenario the virus could arrive in Ghana via migratory birds but also (and perhaps more plausibly) by the arrival in Ghana of infected individuals traveling from other countries.

## Abbreviations

CFR: Case fatality rates
EU: European Union
MDAs: Ministries, Departments, and Agencies
NADMO: National Disaster management Organization
NCC: National Coordinating Committee
NCCE: National Commission on Civic Education
NISPPI: Ghana National Integrated Strategic Plan for Pandemic Influenza
SARS: Severe Acute Respiratory Syndrome
